# Improved artificial intelligence discrimination of minor histological populations by supplementing with color-adjusted images

**DOI:** 10.1038/s41598-023-46472-7

**Published:** 2023-11-04

**Authors:** Satomi Hatta, Yoshihito Ichiuji, Shingo Mabu, Mauricio Kugler, Hidekata Hontani, Tadakazu Okoshi, Haruki Fuse, Takako Kawada, Shoji Kido, Yoshiaki Imamura, Hironobu Naiki, Kunihiro Inai

**Affiliations:** 1 Division of Molecular Pathology, Department of Pathological Sciences, University of Fukui, 23-3 Matsuoka-Shimoaizuki, Eiheiji, Fukui 910-1193 Japan; 2 Division of Diagnostic/Surgical Pathology, University of Fukui Hospital, Eiheiji, Japan; 3 Graduate School of Sciences and Technology for Innovation, Yamaguchi University, Yamaguchi, Japan; 4 Department of Computer Science, Nagoya Institute of Technology, Nagoya, Japan; 5 Department of Pathology, Fukui Red Cross Hospital, Fukui, Japan; 6 Department of Clinical Inspection, Maizuru Kyosai Hospital, Maizuru, Japan; 7 Department of Artificial Intelligence Diagnostic Radiology, Osaka University Graduate School of Medicine, Suita, Japan

**Keywords:** Medical research, Engineering

## Abstract

Despite the dedicated research of artificial intelligence (AI) for pathological images, the construction of AI applicable to histopathological tissue subtypes, is limited by insufficient dataset collection owing to disease infrequency. Here, we present a solution involving the addition of supplemental tissue array (TA) images that are adjusted to the tonality of the main data using a cycle-consistent generative adversarial network (CycleGAN) to the training data for rare tissue types. F1 scores of rare tissue types that constitute < 1.2% of the training data were significantly increased by improving recall values after adding color-adjusted TA images constituting < 0.65% of total training patches. The detector also enabled the equivalent discrimination of clinical images from two distinct hospitals and the capability was more increased following color-correction of test data before AI identification (F1 score from 45.2 ± 27.1 to 77.1 ± 10.3, *p* < 0.01). These methods also classified intraoperative frozen sections, while excessive supplementation paradoxically decreased F1 scores. These results identify strategies for building an AI that preserves the imbalance between training data with large differences in actual disease frequencies, which is important for constructing AI for practical histopathological classification.

## Introduction

Since the last decade of the twentieth century, innovative advances in computer and digitization technologies have initiated methods for the digital storage and utilization of a variety of medical information, especially medical images^1^. Further advances in medical engineering have enabled the usage of medical instruments such as endoscopy^2^, computed tomography^3^, and magnetic resonance imaging^4^. Recent developments have focused on modern computer-aided diagnoses relying on advancements in artificial intelligence (AI) technology. Indeed, AI research is rapidly progressing after the development of the convolutional neural network-based deep learning approach in the fields of radiology^5^, endoscopy^2^, ophthalmology^6^, dermatology^7^, and pathology^8^ because of its high affinity to medical images.

Pathology has become methodologically systematized based on investigating grossly and/or visually identified abnormal lesions representing morphological evidence in human bodies^9–12^. Pathomorphological diagnosis is performed using hematoxylin–eosin (HE) stained samples of thin paraffin-embedded tissue sections mounted on glass slides. Recently, the development of digital devices, including charge coupled device cameras and virtual slide systems, has enabled the digital preservation of multi-colored histopathology images. Utilization of these images has progressed to histopathological AI investigations due to the widespread dissemination of virtual slide systems. Histopathological AI explores diverse fields including tumor classification, detection of metastases^13^, prediction of tumor antigens^14^ and gene mutations^15, 16^ as alternatives to immunohistochemistry and genomic retrieval, as well as prediction of therapeutic responses^17^ and survival rates^18^. In the future, AI is expected to provide benefits to pathologists in the areas of diagnostic support and correction of diagnostic disparities between facilities and between diagnosticians. However, the current applications available for clinical pathology remain under development.

In the diagnostic process of clinical medicine, pathologists as well as physicians consider the frequency of disease based on epidemiological data. Namely, medical doctors seldom consider the presence of rare diseases or histologically rare cancers at the beginning of the examination. In contrast, AI investigations were initiated as a binarization task for tumor classification between benign or malignant lesions^19^, followed by the rough discrimination of adenocarcinoma and squamous cell carcinoma^20^. At present, more detailed classification of tissue subtypes is being investigated^21–27^; however, most efforts are limited to simply increasing the broad kinds of histologic subtypes. The development of immunohistochemical staining and genetic analyses have accelerated the tissue subdivision of malignant tumors. In fact, the WHO classification recognizes hundreds of tissue types in each organ^28, 29^. Although detailed characterizations of malignancies provide important information for personalized medicine, the expanded occurrence frequency of each histological type becomes a limitation for obtaining sufficient training images for deep learning.

Large imbalances among training datasets reduce the performance of AI. Many research institutions conduct multicenter studies, and/or utilize the released digital image atlases, such as The Cancer Genome Atlas^21, 30, 31^, in order to obtain sufficient data. While these worldwide collaborations are useful, they present a risk of disparity in image quality due to the utilization of non-standardized procedures for sample preparations and staining methods, thereby causing uneven coloration, as well as different image capture conditions, and deterioration between specimens. Moreover, equalization of the number of training data takes precedence over the actual frequency of disease. As a result, it is difficult for histopathological AI to learn rare tissue types, which is essential for clinical applications; moreover, classification into a limited number of histologic types has rarely been considered in present histopathology AI studies. Thus, in this study, we tried to establish an AI that followed objective epidemiological data and the thought processes of medical doctors as end-user, rather than comparing the superiority or inferiority of AI-programs or theories.

**Figure 1:**
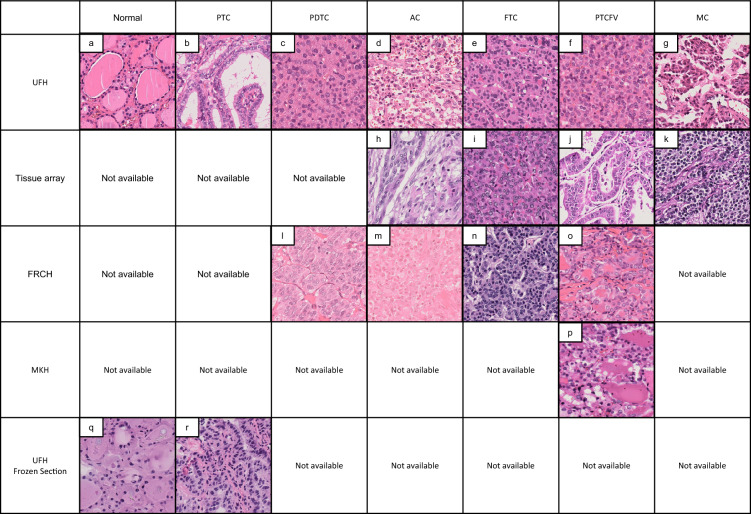
Representative histological images of thyroid cancer tissues from different sources. This is a representative image of HE specimens from each institution. Even with the same tissue type, the appearance differs depending on the difference in staining. AC, anaplastic carcinoma; FRCH, Japanese Red Cross Fukui Hospital; FS, frozen section; FTC, follicular thyroid carcinoma; MC, medullary carcinoma; MKH, Maizuru Kyosai Hospital; PDTC, poorly differentiated thyroid carcinoma; PTC, papillary thyroid carcinoma; PTCFV, papillary thyroid carcinoma, follicular variant; TA, tissue microarray; UFH, University of Fukui Hospital.

**Figure 2:**
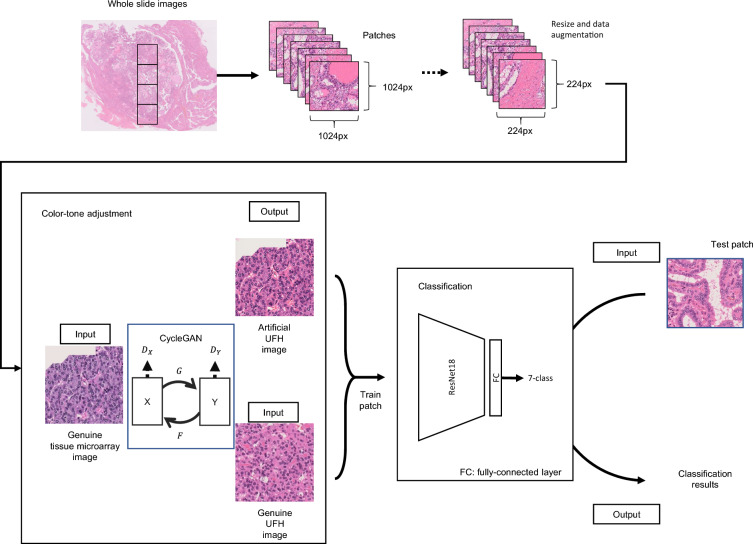
Schematic model for the color-tone adjustment using CycleGAN and the construction of the classifier using ResNet18. Tumor lesions in whole slide images were manually annotated by pathologists and the images were subsequently exported and cropped into patches. Resize and data augmentation were applied to the patches and the patches were used to train CycleGAN. The ResNet18-based classifier was trained on the patch images including color-tone adjusted images. In CycleGAN, four neural networks were used: generators G and F, and discriminators Dx and Dy. In this study, X and Y represent the tissue microarray image domain and the UFH image domain, respectively. Abbreviations: G, a generator that transforms X to Y; F, a generator that transforms Y to X; Dx, a discriminator that discriminates X from F(Y); Dy, a discriminator that discriminates Y from G(X). Abbreviations: TA, tissue array; UFH, University of Fukui Hospital.

Recently, it was reported that maintaining the imbalance between the largest and smallest number of training datasets, up to 50-fold disparity, improves histopathological AI investigations^23^. Additionally, the development of cycle-consistent adversarial networks (CycleGAN), an unsupervised image transformation network model, has made it possible to create images with matching qualities such as color tone^32^. These circumstances let us hypothesize that a collaborative medical-engineering research team could construct an AI system that would enable tissue subclassification while preserving the disease frequencies observed in clinical practice. In this study, we explored a rational method to complement a small number of histological type images in a thyroid cancer model that mimicked the approximately 85-fold difference in disease frequency observed in clinical practice^33^. Herein, we show that supplementation of color-tone adjusted minor tissue subtypes, about 1% of all training patches produced by CycleGAN, from tissue array images dramatically improves the discrimination ability of histopathological AI (Figs. 1, 2).

## Results

### Poor discriminative effect of the classifier established from the original HE images only

We first established an initial classifier to ascertain the ability of ResNet18 using the HE-stained original UFH patches. In the patch sets, imbalances of patch numbers were documented as 109,442 (81.71% of total patches) of PTC, 8353 (6.24%) of Normal, 6537 (4.88%) of AC, 5223 (3.90%) of PDTC, 1597 (1.19%) of MC, 1425 (1.06%) of FTC, and of 1360 (1.01%) of PTCFV, respectively (Table 1). More than 80% of the training patches were occupied by PTC with an 80 × enrichment relative to the minimum of PTCFV. The classifier could well-classify the four histological types (PTC, Normal, AC, and PDTC) with recall values between 97.5 and 64.7, precision between 95.5 and 78.1, and F1 scores between 96.5 and 74.3%, while the parameters for the remaining three neoplasms were dramatically lower (Table 1).

**Table 1:** Training patch number and classification result of initial experiment.

	Normal	PTC	PDTC	AC	FTC	PTCFV	MC
Training patches	8353	109,442	5223	6537	1425	1360	1597
% of training patches (magnification)	6.24 (× 6.12)	81.71 (× 80.11)	3.90 (× 3.82)	4.88 (× 4.78)	1.06 (× 1.04)	1.02 (× 1)	1.19 (× 1.17)
F1 score	92.3	96.5	86.4	74.3	25.5	28	19.5
Precision	87.7	95.5	78.1	87.2	59	31.6	81.4
Recall	97.4	97.5	96.6	64.7	16.3	25.1	11

AC, anaplastic carcinoma; FTC, follicular thyroid carcinoma; MC, medullary carcinoma; PDTC, poorly differentiated thyroid carcinoma; PTC, papillary thyroid carcinoma; PTCFV, papillary thyroid carcinoma, follicular variant.

### Effectiveness of adding a small number of color-tone corrected tissue images to training data

The imbalance of training patches among histological subtypes was considered a reason for detector malfunction, whereas the supplementation of training images is not a simple task because of the inherent histological infrequencies. Additionally, the tissue collection methods, duration of formalin fixation, and/or the composition of staining solutions is a source of variability among facilities; thus, it is essential to not only collect new tissue images, but also unify the color tones in order to resolve the heterogeneities among multisource sample conditions preserving the actual histological frequency according to the pathologists' thought processes. Thus, color-tone adjustment was implemented in CycleGAN. The color tone-adjustments were evaluated to be suitable levels for pathological diagnoses by nine pathologists (data not shown).

### Optimal classifier construction following addition of color-adjusted training images

**Figure 3:**
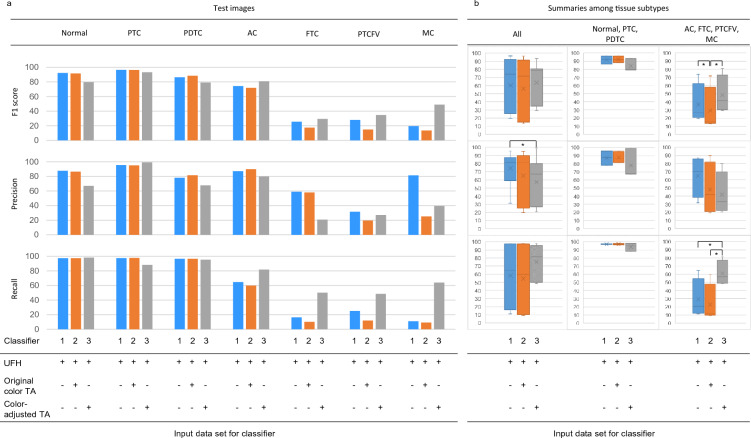
Discriminative effects of supplementing with color-adjusted TA images on original UFH images. (**a**) F1 score, precision, and recall for each tissue subtype. Three different conditions of classifiers are shown below the bar chart. Classifiers 1, 2, and 3 are represented by the blue, orange, and grey bars, respectively. (**b**) Boxplot summaries among the tissue subtypes. The left column shows the boxplot summaries of F1 score, precision, and recall from all tissue subtypes. The middle and left columns present the detailed results stratified into Normal, PTC to PDTC data, and AC, FTC, PTCFV to MC data, respectively. AC, anaplastic carcinoma; FTC, follicular thyroid carcinoma; MC, medullary carcinoma; PDTC, poorly differentiated thyroid carcinoma; PTC, papillary thyroid carcinoma; PTCFV, papillary thyroid carcinoma, follicular variant; TA, tissue array. *p < 0.05.

To optimize the classifier in the presence of variations in the distribution of training data, small numbers of imaging patches with or without color processing were appended to the original UFH training data, and the discriminative effects were evaluated. Compared with classifier 1, composed entirely of UFH training data, concurrent addition of original TA-patches in the classifier 1 data set (classifier 2) maintained the same F1 score, precision, and recall values as those in classifier 1. Moreover, the levels in FTC, PTCFV, and MC had a tendency to be worse than those in classifier 1. In contrast, classifier 3, with color-adjusted TA patches replacing the original TA images, dramatically improved the recall values in FTC, PTCFV, and MC, which in turn increased the F1 scores (Fig. 3a). In Fig. 3b, the data were re-evaluated using a box plot. Overall, the F1 score due to classifier 3 was significantly increased relative to those of both classifier 1 and classifier 2 (*p* < 0.05, N = 4), which was attributable to the dramatic increase in recall value (*p* < 0.05, N = 4), especially among the four minor tissue types whose AI evaluations before color-tone adjustment had exhibited poor scores. Although classifier 3 had lower precision than classifier1 across histological subtypes (*p* < 0.05, N = 7), there was no significant difference in recall and F1 scores.

**Figure 4:**
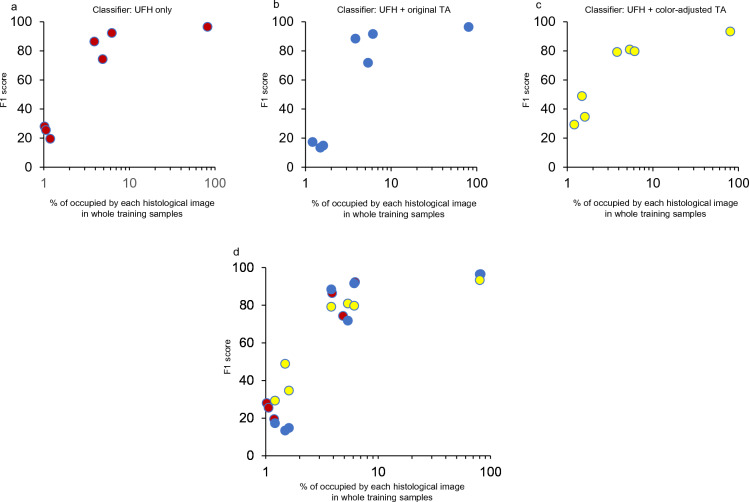
The relationship between F1 score and the percentage occupied by each histological subtype. The F1 scores for the three discriminators were plotted in (**a**–**c**), and the overlaid image is shown in (**d**). TA, tissue microarray; UFH, University of Fukui Hospital.

To verify the benefits of supplementing patch images with or without color-adjustment, we further investigated the association between F1 score and the percent occupied in each histological image in whole training samples. While supplementation with only 1% color-corrected TA images improved the discriminator's ability for minor tissue populations, the addition of the original unprocessed images worsened the ability relative to the original classifier (Fig. 4a–d).

### Color-tone adjustments of both training and test data further improves the discrimination capability of a small number of clinical images

The addition of color-adjusted training data facilitated identification of minor thyroid cancers (< 2%) in the clinical setting. These results allowed the investigation of discrimination potency with color-tone corrections for the establishment of a classifier as well as for a small numbers of test data derived from rare tissue types and/or from few specimens submitted by clinical hospitals.

**Figure 5:**
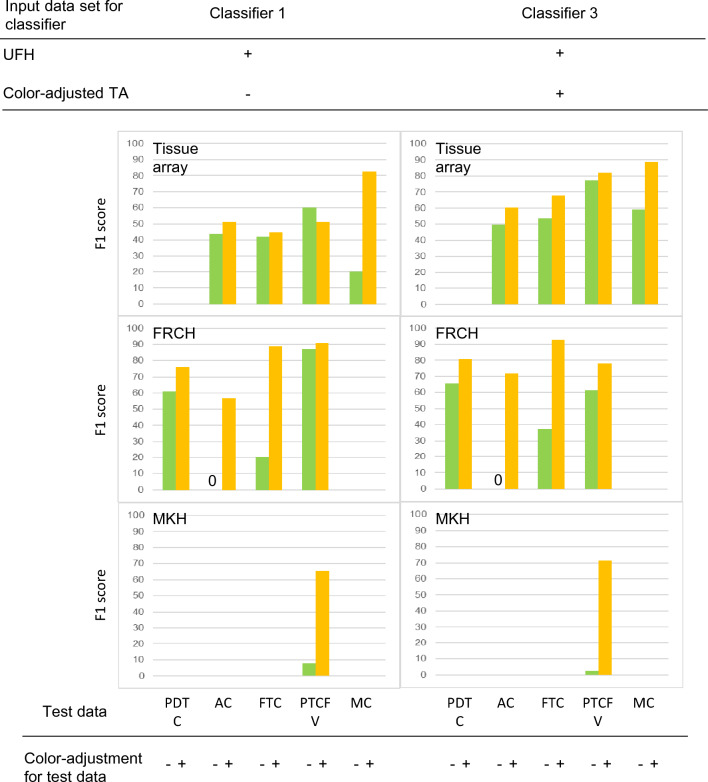
Effect of color-tone adjustments of both training and test data on F1 scores in a small number of clinical images. The F1 scores of minor tissue types of TA and two clinical hospitals (FRCH and MKH) were classified in the presence or absence of color-tone adjustment of both training and test data. Abbreviations: AC, anaplastic carcinoma; FRCH, Japanese Red Cross Fukui Hospital; FTC, follicular thyroid carcinoma; MC, medullary carcinoma; MKH, Maizuru Kyosai Hospital; PDTC, poorly differentiated thyroid carcinoma; PTCFV, papillary thyroid carcinoma, follicular variant; TA, tissue microarray; UFH, University of Fukui Hospital.

The F1 scores of minor tissue types of TA and two clinical hospitals (FRCH and MKH) are shown in Fig. 5. In the identification of conventional test data (green bar), the F1 scores for classifier 3 tended to be higher than those for classifier 1, except for PTCFV of FRCH. The color-corrected test data were then classified by classifier 1 or classifier 3. The results showed that the identification results for the pre-processed test data (yellow bar) were superior to those of the non-color-corrected data (green bar) using both classifiers. Notably, the F1 scores of the color-corrected specimens from classifier 3 were higher than those without color correction for all tissue types submitted from TA, FRCH, and MKH.

**Figure 6:**
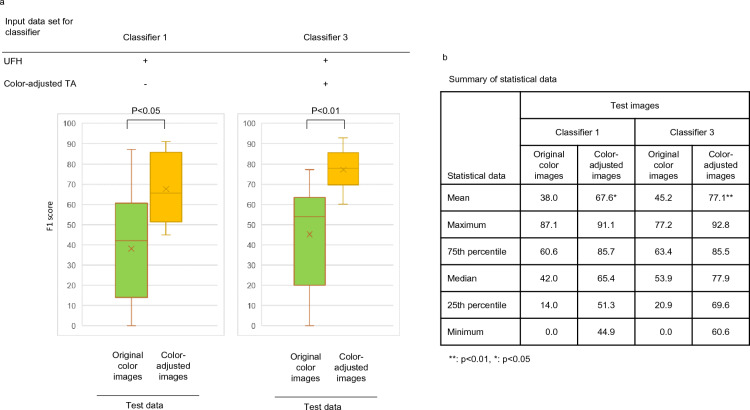
Changes in F1 score of color-tone adjustments in the presence or absence of both training and test data in a small number of clinical images. Box plots and their raw statistical data are shown in (**a**) and (**b**), respectively. TA, tissue microarray; UFH, University of Fukui Hospital.

Summarizing the above data using box plots, the color-adjusted minor test images from TA, FRCH, and MKH exhibited significantly improved F1 scores using both classifier 1 (*p* < 0.05, N = 9) and classifier 3 (*p* < 0.01, N = 9) relative to the original test data (Fig. 6a and b), indicating that color-tone adjustment of both training and test data is recommended for classifying minor tissue populations by histopathological AI.

### Adaptation of color-tone adjustment for images derived from frozen tissue sections

**Figure 7:**
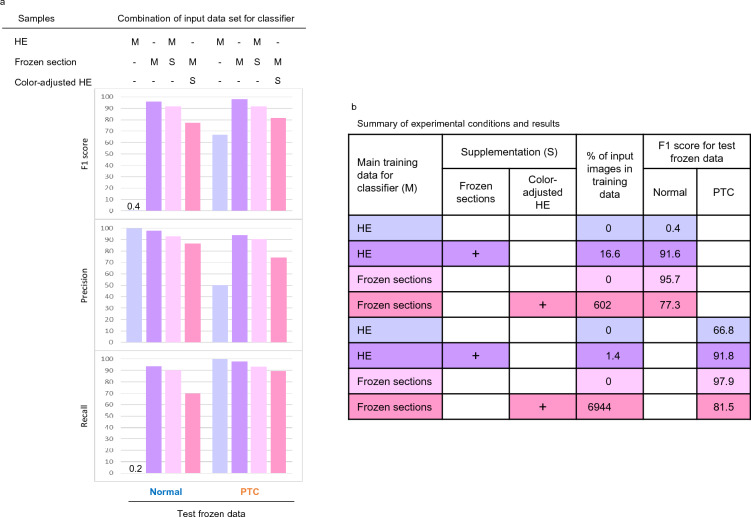
Adaptation of color-tone adjustment for the images derived from intraoperative frozen tissue sections. (**a**) F1 score, precision, and recall of Normal and PTC frozen images by each classifier. (**b**) Summary of experimental conditions and the respective F1 scores. Abbreviations: HE, hematoxylin and eosin; M, main training data; PTC, papillary thyroid carcinoma; S, supplementation.

Intraoperative frozen sections (FS) are an alternative method for rapid diagnosis during surgical procedures, which are performed not only in-hospital but also between affiliated hospitals such as regional family doctors, community medical support hospitals, and an advanced treatment hospital using telepathology technique^34^. FSs are also stained by HE dyes; however, the tissue preparation and staining methods differ from those in conventional paraffin-embedded samples between the hospitals. Therefore, we examined whether the color-adjustment adaptations were beneficial in establishing a classifier for FS. In this experiment, four classifiers were applied to the images with the original HE only, FSs only, original HE plus supplemental FSs, or FSs supplemented with color-tone corrected HE (Fig. 7a). The classifiers constructed with only FSs with 100% of the input images (purple) represented the highest F1 scores for discriminating both Normal (F1 score: 95.7) and PTC (97.9). In contrast, the detectors established with the original HE images only (light blue) exhibited reduced F1 scores, with an especially dramatic decline for Normal (0.4). Interestingly, the discriminative ability increased dramatically in Normal (91.6) and PTC (91.8) when 16.6 or 1.4% of FS images were added to the discriminator consisting of HE images only (pale pink). On the other hand, discriminators composed of FSs only, which had the highest discriminative power, showed relatively poor discriminative ability due to the addition of 60 (Normal) to 700 (PTC) fold more color-altered images (deep pink) than the main FSs (Fig. 7b).

## Discussion

Histopathological AI has surpassed first generation binary classification^19^, second generation cancer tissue classifications^20^, and is now in the third generation of tissue discrimination^21–27^. However, AI classification has not reached the capability of providing the detailed tissue classifications required in clinical practice. In the present study, we initially created a discriminator for thyroid cancer tissues using histological patch images from our facility and found that a large difference in the F1 scores emerged among eight tissue types (from 19.5 to 96.5). Notably, training data with low scores represented about 1% of cases in all training datasets, indicating that imbalances among histological types caused large differences. With our protocol, we had already augmented rotated and/or flipped the original images in order to establish an initial classifier. However, the number of patches for several tissue types remained insufficient, and commercially available TAs were needed to increase the number of patches for rare tissue types. Unfortunately, the addition of these images had little effect on the ability to discriminate tissue types using the original training data. In contrast, the recall, precision, and F1 scores of rare histological types were decreased following the addition of TA images to the original training dataset. This observation suggests that simple supplementation with the lacking image data is insufficient to optimize the detector.

Thus, image quality as well as the number of images represents a possible alternative factor for improving detector accuracy. At present, standardized conditions for machine learning identification of HE-stained tissue images haven’t been established yet, as many combinations of contrast, brightness, and/or hue/saturation/warmth of color can be assumed. Therefore, in this study, we focused on converting the same TA image used to create the initial classifier to the color tones of the original UFH image using CycleGAN. The adjusted images were then used to supplement the training dataset prior to reconstruction of the classifier. Although the percentage of TA images with additional tinting was only 1.7% of the total training patches, this operation significantly enhanced the recall value, resulting in a significantly increased F1 score. The effect of color tone correction became particularly evident for tissue types with little training data, suggesting that the number of quantitative trained images as well as their image qualities had substantial influence on the configuration of the detector. Furthermore, the usage of color-tone adjusted training data also benefited the discrimination of tissue images from several clinical hospitals. This result was thought to be due to possessing redundancy of the tonal characteristics of various tissue images from different facilities due to the addition of color-tone corrected TA images by CycleGAN to the main training data. More interestingly, the highest discrimination level was achieved when not only the a few training TA data but also the test images were color-corrected with CycleGAN to a UFH-like color tone prior to discrimination. These findings are suggested to provide a benefit for the creation and using of practical discriminators for AI diagnosis of pathology images from clinical facilities.

Intraoperative FS for histopathological diagnosis differs from conventional paraffin sections in the preparation procedures, including the tissue fixative method and fixation time. Additionally, histopathological diagnosis using intraoperative FS is conducted less frequently than with paraffin sections, which indicates that constructing a detector for intraoperative rapid diagnosis is more difficult. Therefore, we investigated whether the ability of CycleGAN to add a small number of color-corrected FS images to HE stains could improve the classification results of FS images compared to training with HE images alone. Specifically, 16.6% and 1.4% of each FS with color correction were added to the training dataset and discriminators was established using HE-based Normal and PTC images. The resultant F1 scores were 91.6 and 91.8, which represented dramatic increases from values of 0.4 and 66.8 produced by the detectors trained only with HE images. Conversely, when a small number of FS images were used as the training images and the insufficient data were supplemented with numerous HE images tonally changed to FS, the F1 score was worse than that of the detector constructed using only FS images. This result suggested that supplementing the training dataset with large amounts of color-corrected images was not capable of resolving the classification issue, but rather that an optimal supplementation ratio exists for the main dataset.

In the field of deep learning-based AI, classification of imbalanced training datasets is known as an important problem^35, 36^. Histopathological AI lacks practicality unless detailed tissue subtypes can be classified by the discriminator. However, in correcting for data imbalance, it is not clear how closely the frequency of tissue subtypes in training data can reflect observed disease frequencies. Among thyroid cancers, the most common PTCs compose 85% of all thyroid cancers, with some histological types occupying less than a few percent^33^. Similar imbalances among tissue types are observed in several tumors, for example B-cell lymphomas have 51 histological types (according to “Tumours of Hematopoietic and Lymphoid Tissues”; WHO classification) yet 60% of the malignancies are accounted for by the top 2 (follicular lymphoma and diffuse large B-cell lymphoma)^29^. Even in the deadliest lung cancer in the world, rare histological adenocarcinomas are present in only about 1% of all lung cancers, and there remain some tissue subtypes with unknown incidence frequencies^28^. In this study, the largest number of PTCs accounted for 81.7% of cases in the training data, a ratio that is relatively close to the clinical epidemiology of thyroid cancer. Moreover, there was an approximately 80-fold difference in training data between the three tissue types. Nevertheless, the F1 score was significantly improved after adding a small number of color-adjusted TA images.

Histopathological AI investigations targeting tissue subtypes have increased in recent years^21–27^. Concerning the composition of the training data in these papers, the mean percentages of training data with the most and least frequent tissue types were 30.3 ± 8.1% (22.6–44%, 95% CI 20.2–40.4%) and 7.7 ± 6.2% (1.1–16.2%, 95% CI 0.0–15.4%), respectively, with a disparity of 9.8 ± 10.1 fold (1.8–21.2 fold, 95% CI − 2.6–21.2 fold)^21–27^. Compared with those manuscripts, this study was capable of expanding this gap from a minimum of 1% to a maximum of 81.7%, ratios that are relatively close to the clinical epidemiology of thyroid cancer. Moreover, there was an 80.1-fold difference in tissue types among the training datasets. Nevertheless, the F1 score was significantly improved after adding a small number of color-adjusted TA images using CycleGAN. As development of various AI for histopathological diagnosis continues to accelerate in the near future, the more we will encounter the problem of imbalances among training datasets in implementing practical applications. Therefore, our method can be utilized as a practical solution to resolve the imbalance in the composition of training data.

This research has several limitations. First, since the detector was mainly constructed based on single center images, the detection accuracy with training data from multiple facilities has not been verified. Second, the target disease of this study was limited to thyroid cancer. Therefore, it is unclear whether similar results could be obtained in other cancer tissues. Thirdly, we estimated the suitable addition ratio of the color tone-corrected images as from 1.5% to 20% of the main training data; however, further examination will be needed to determine optimal conditions. Fourth, there is no unanimous opinion regarding what F1 values should be considered effective, although several documents consider an effective level of F1 to be > 70^21, 23, 37^. The purpose of this study was to clarify the effectiveness of color-tone correction in order to establish an AI that is consistent with the pathologist's thought process in terms of disease frequency. Thus, we did not compare our results of CycleGAN with those using other systems such as over samplings, under samplings, cost sensitive trainings, and a state-of-art diffusion model^38^ as a generative AI model, that was considered as the fifth limitation of this study.

In conclusion, we conducted an AI construction study for tissue subtype classification using thyroid cancer preserving epidemiological interclass imbalance and found that the utilization of a small number of color-corrected images using CycleGAN for different resource images was useful for improving the imbalance in the number of training data. Recently, the management of the color tone of the tissue samples in the pathological AI construction has become so important that the International Color Consortium has been formed and discussed for this purpose ^39, 40^. Therefore, further studies will be needed, we concluded that this research highlighted important strategies for constructing AI for practical histopathological classification.

## Materials and methods

### Sample collection, preparation, and diagnoses of thyroid tissues

Human thyroid tissues subjected to surgical resection at University of Fukui Hospital (UFH), an advanced treatment hospital, were used in this study. Whole thyroid glands and partially resected samples were quickly soaked in 10% formalin solution. The fixed tissues were cut into sections 5 mm in width, embedded in paraffin, and thinly sliced into 4 μm sections, followed by staining with pre-conditioned HE solution (Hematoxylin3G and Eosin, Sakura Finetek, Tokyo, Japan) using a VENTANA HE600 automatic staining apparatus (Roche Diagnostics, Tokyo, Japan) in our Department of Surgical Pathology laboratory.

In patients where the existence of malignancy was suspected but not confirmed following pre-operative inspections, intraoperative pathological analysis was performed using frozen tissue sections. Briefly, an approximately 5 mm square of the suspected lesion was embedded in OCT compound (Yuaikasei, Hyogo, Japan) and the tissue was quickly frozen for 1 min at − 85 °C in the presence of Histo-Tek Hyfluid refrigerant (Sakura Finetek, Tokyo, Japan). Following the preparation of thinly sliced frozen tissue, the sections were adhered to a glass slide and soaked in 100% ethanol for fixation. The fixed section was then stained by HE dye in the hospital lab.

All histological diagnoses were performed by board-certified pathologists and pathology trainees as follows: normal region, papillary thyroid carcinoma (PTC), papillary thyroid carcinoma follicular variant (PTCFV), follicular thyroid carcinoma (FTC), poorly differentiated thyroid carcinoma (PDTC), medullary carcinoma (MC), anaplastic carcinoma (AC) and other benign diagnoses were according to the criteria of the 4^th^ edition of the WHO classification of head and neck tumors^41^.

In accordance with clinical research guidelines, the Ethics Committee approved that written consent from the patient was not required because this study was used only histopathology images with no risk of identifying personal information.

### Data processing and digitalization

The HE-stained slide section (150) were selected from the previous post-operative thyroid cancer tissues at UFH. The normal thyroid region apart from the tumor area on the same slide in PTC was used as the normal thyroid tissue (Normal). Intraoperative frozen images (frozen section: FS) were also included in this experiment in order to analyze the influence of different preparations. To supplement the dataset with images of rare histological subtypes, commercially available undyed tissue microarrays (TA) were purchased from Funakoshi (Tokyo, Japan). The HE-staining of TA slides was performed in a laboratory of the Division of Molecular Pathology. Clinical samples from two different community medical support hospitals, Japanese Red Cross Fukui Hospital (FRCH) and Maizuru Kyosai Hospital (MKH), were prepared.

Digitalization of whole slide images (WSIs) was performed using a NanoZoomer S60 digital slide scanner (Hamamatsu Photonics, Hamamatsu, Japan). Briefly, the whole area of each tissue slide was captured at 40 × magnification and stored on a computer. The WSIs were exported in TIFF format and the manually annotated tumor areas were extracted as regions of interest (ROIs) and cropped into patches of 1024 × 1024 pixels for model development. Representative paraffin (Fig. 1a–p) and frozen (Fig. 1q and r) patch images are shown in Fig. 1, and the number of glass slides and their patches are summarized in Supplementary Table S1. The images were divided into two groups for training and test sets at a 2:1 ratio.

All research protocols were approved by the ethics review board at the University of Fukui Hospital and conformed to the provisions of the Declaration of Helsinki. Approval for an opt-out method was also given by the ethics committee.

### Deep learning network

We fine-tuned ResNet18 pre-trained with ImageNet. ResNet18 is a popular convolutional neural network that uses residual connections. The network structure for fine-tuning consists of the feature extraction layers of ResNet18, a dropout layer that randomly invalidates 40% of the connections, and a fully connected layer that outputs the classification results. The patches to input to the network were resized to 224 × 224 pixels considering the memory constraints of the graphics processing unit (GPU). For data augmentation, random rotation of 0, 90, 180, 270 degrees, up, down, left and right inversion, standardization using the mean and standard deviation of pixel values of all the ImageNet images were applied to the patch images. In order to maintain the data balance of the original images in each histology, the original image data used for color conversion was eliminated from the dataset when using the artificial images synthesized by CycleGAN.

The loss function is cross entropy, the optimization method is Adam, and the parameters related to the training were set as follows. The learning rate of the feature extraction layer was set to 0.00001, that of the fully connected layer was 0.0001, and the weight decay was 0.0001. The batch size was 32 and the number of epochs was 10. The flow of making patches, data augmentation, and classification using the classifier combining ResNet18 and a fully-connected layer is shown in Fig. 2.

### Digital color-adjustment by CycleGAN

We used CycleGAN for adjusting the color tone of HE of images from TA, FRCH, MKH, and FS. The structure of CycleGAN is shown in lower left box in Fig. 2, where X is a domain of tissue microarray images and Y is that of UFH images. CycleGAN can learn the features of X and Y, and finally it can transform X to Y-like images. In this study, this transformation realizes color-adjustment from tissue microarray images to UFH images. The left genuine tissue microarray image shows an example of an input tissue microarray image and the upper right image shows the result of color-adjustment. The lower right image is a genuine UFH image; therefore, we can see that the tissue microarray image is transformed to a UFH-like color image. The color tone conversion of FRCH, MKH, and FS was also carried out using the same methods.

### Statistical analysis

BellCurve for Excel software ver. 4.01 (Social Survey Research Information, Tokyo, Japan) was used for statistical analysis. Two-tailed test with *P* value < 0.05 were accepted as statistically significant. BellCurve was used to evaluate the performance of the classifier, recall, precision, and F1 scores.

### Ethical approval

All research protocols were approved by the ethics review board at the University of Fukui Hospital (No. 20200084) and conformed to the provisions of the Declaration of Helsinki. In accordance with clinical research guidelines, the committee approved that written consent from the patient was not required because this study was used only histopathology images with no risk of identifying personal information.

### Supplementary Information

Supplementary Table 1.

## Data Availability

The de-identified data (patch images) used in this study are available on request from the corresponding author. The original image data are not publicly available because they contain patient information.

## Code availability

Since the code is specialized for this problem, it is difficult to publish it.
