# Supplementary material for: Improved artificial intelligence discrimination of minor histological populations by supplementing with color-adjusted images
Source: Sci Rep. 2023 Nov 4;13:19068. doi: 10.1038/s41598-023-46472-7 (PMC10625567; doi:10.1038/s41598-023-46472-7)
Supplement: Supplementary file 1 — Supplementary Table 1. [file 41598_2023_46472_MOESM1_ESM.pdf]

Supplementary Table S1. Numbers of patch images and the purpose of their use

		Morphological diagnosis							Purpose of use		
		Normal	PTC	PDTC	AC	FTC	PTCFV	MC	ResNet18	CycleGAN	FS
UFH	slides	7	76	9	12	7	34	5			
	total	11690	146023	2871	10594	2962	1859	2113			
	training	8353	109442	5223	6537	1425	1360	1597	Yes	Yes	Yes
	test	3105	33175	1901	2228	503	462	516			
TA	slides*				34	67	9	37			
	total	0	0	0	1056	301	1133	580			
	training	0	0	0	785	215	830	433	Yes	Yes	
	test	0	0	0	271	76	303	147			
FRCH	slides			2	2	2	2				
	total	0	0	339	334	350	345	0			
	training	0	0	172	168	192	182	0		Yes	
	test	0	0	159	166	158	163	0			
MKH	slides						2				
	total	0	0	0	0	0	248	0			
	training	0	0	0	0	0	176	0		Yes	
	test	0	0	0	0	0	72	0			
FS	slides	10	8								
	total	2237	2084	0	0	0	0	0			
	training	1664	1599	0	0	0	0	0			Yes
	test	573	485	0	0	0	0	0			

* The number of captured core images on tissue microarray slides for this study. Usually, 15 to 20 patches were cropped from each core.

Abbreviations: AC, anaplastic carcinoma; FRCH, Japanese Red Cross Fukui Hospital; FS, frozen section; FTC, follicular thyroid carcinoma; MC, medullary carcinoma; MKH, Maizuru Kyosai Hospital; PDTC, poorly differentiated thyroid carcinoma; PTC, papillary thyroid carcinoma; PTCFV, papillary thyroid carcinoma, follicular variant; TA, tissue microarray; UFH, University of Fukui Hospital.
